# A Pan-Cancer Analysis Revealing the Dual Roles of Lysine (K)-Specific Demethylase 6B in Tumorigenesis and Immunity

**DOI:** 10.3389/fgene.2022.912003

**Published:** 2022-06-14

**Authors:** Jia-Tong Ding, Xiao-Ting Yu, Jin-Hao He, De-Zhi Chen, Fei Guo

**Affiliations:** ^1^ Ningbo Institute for Medicine & Biomedical Engineering Combined Innovation, Ningbo Medical Centre Lihuili Hospital, Ningbo University, Ningbo, China; ^2^ The Second Clinical Medical College of Nanchang University, Nanchang, China; ^3^ Burn Research Institute, The First Affiliated Hospital of Nanchang University, Nanchang, China

**Keywords:** KDM6B, tumorigenesis, prognosis, immunotherapy, epigenetic regulation

## Abstract

**Introduction:** Epigenetic-targeted therapy has been increasingly applied in the treatment of cancers. Lysine (K)-specific demethylase 6B (KDM6B) is an epigenetic enzyme involved in the coordinated control between cellular intrinsic regulators and the tissue microenvironment whereas the pan-cancer analysis of KDM6B remains unavailable.

**Methods:** The dual role of KDM6B in 33 cancers was investigated based on the GEO (Gene Expression Omnibus) and TCGA (The Cancer Genome Atlas) databases. TIMER2 and GEPIA2 were applied to investigate the KDM6B levels in different subtypes or stages of tumors. Besides, the Human Protein Atlas database allowed us to conduct a pan-cancer study of the KDM6B protein levels. GEPIA2 and Kaplan–Meier plotter were used for the prognosis analysis in different cancers. Characterization of genetic modifications of the KDM6B gene was analyzed by the cBioPortal. DNA methylation levels of different KDM6B probes in different TCGA tumors were analyzed by MEXPRESS. TIMER2 was applied to determine the association of the KDM6B expression and immune infiltration and DNA methyltransferases. Spearman correlation analysis was used to assess the association of the KDM6B expression with TMB (tumor mutation burden) and MSI (microsatellite instability). The KEGG (Kyoto encyclopedia of genes and genomes) pathway analysis and GO (Gene ontology) enrichment analysis were used to further investigate the potential mechanism of KDM6B in tumor pathophysiology.

**Results:** KDM6B was downregulated in 11 cancer types and upregulated across five types. In KIRC (kidney renal clear cell carcinoma) and OV (ovarian serous cystadenocarcinoma), the KDM6B level was significantly associated with the pathological stage. A high level of KDM6B was related to poor OS (overall survival) outcomes for THCA (thyroid carcinoma), while a low level was correlated with poor OS and DFS (disease-free survival) prognosis of KIRC. The KDM6B expression level was associated with TMB, MSI, and immune cell infiltration, particularly cancer-associated fibroblasts, across various cancer types with different correlations. Furthermore, the enrichment analysis revealed the relationship between H3K4 and H3K27 methylation and KDM6B function.

**Conclusion:** Dysregulation of the DNA methyltransferase activity and methylation levels of H3K4 and H3K27 may involve in the dual role of KDM6B in tumorigenesis and development. Our study offered a relatively comprehensive understanding of KDM6B’s dual role in cancer development and response to immunotherapy.

## Introduction

Epigenetic dysregulation leads to aberrant transcriptional programs that promote tumor occurrence and progression (Hogg et al., 2020). Thus, epigenetic therapies focus on normalizing the malignant phenotype-related DNA methylation status or post-translational modifications on histones, exerting their effects as tumor suppressors (Bates, 2020). Enzymes that regulate the methylation of lysine-rich N-terminal histone “tails” contribute to the epigenetic modulation of tumor-associated pathways, such as NF-κB and p53 signaling pathways (Agger et al., 2009; Park et al., 2016). Moreover, epigenetic modification, including histone methylation, has also been identified as one of the mechanisms that silence gene expression and then evade immune cells in solid tumor cells (Ratnam et al., 2021). Therefore, it is necessary to investigate the potential mechanisms of histone methylation regulatory enzymes in pan cancers.

Lysine-specific demethylase 6B (KDM6B) or Jumonji domain-containing protein-3 (JMJD3), belonging to histone H3 lysine 27 (H3K27) demethylases, regulates the gene expression by histone demethylation in response to an intracellular or extracellular stimulation under physiological or pathological conditions. KDM6B regulates a wide range of pathways involved in development, inflammation, and specifically cancers, such as WNT, NF-κB, and BMP (Salminen et al., 2014; Xu et al., 2014; Park et al., 2016). Over-expression of KDM6B results in unhindered transcription and disrupts core gene-regulatory architecture by decreasing the H3K27me3 level, which plays a dual role in cancers (Lagunas-Rangel, 2021). For example, KDM6B serves as an oncogene in pancreatic ductal adenocarcinoma cells *via* demethylating H3K27me3 to prompt the expression of cyclin D1 cooperating with smad2/3 (Cao et al., 2021). However, KDM6B inhibits tumorigenesis *via* removing H3K27me3 to induce neuronal differentiation in neuroblastoma (Yang et al., 2019). Nevertheless, despite the extensive clinical statistics, there is no available cross-sectional evidence of the correlation of KDM6B and different cancers.

Considering the potential link between genes and cancers, it is necessary to explore the correlation between genes of interest and the occurrence and prognosis in pan cancers and the underlying molecular mechanisms. The publicly funded Cancer Genome Atlas (TCGA) project and the Gene Expression Omnibus (GEO) database contain functional genomic datasets for different types of tumors (Sidaway, 2017; Alexandrov et al., 2020; Lee et al., 2020), which makes it possible to perform a pan-cancer analysis. In this article, we reviewed the experimentally determined evidence for the correlation between KDM6B and different cancer types or stages. A pan-cancer study of KDM6B using the TCGA project and the GEO database was conducted here for the first time. We also explored the potential KDM6B-associated pathways in the pathogenesis or clinical outcomes in numerous cancers, including expression difference, survival conditions, genetic changes, DNA methylation, immune infiltration, and related biological processes. Our goal was to determine how KDM6B influences the clinical outcomes and immune infiltration in certain cancers and recognize the potential pathways to offer a relatively comprehensive understanding of KDM6B’s dual role, which may serve as a new therapeutic target.

## Materials and Methods

### Analysis of Gene Expression

We used the TIMER2.0 (Tumor Immune Estimation Resource 2nd Edition) website (http://timer.cistrome.org/) (Li et al., 2020) to detect the difference of the KDM6B expression between tumor tissues and their adjacent normal tissues of various tumors from the TCGA database with the Wilcoxon test except the cancers with extremely restricted or without normal tissues, such as TCGA–DLBC (diffuse large B-cell lymphoma) and TCGA–TGCT (testicular germ cell tumors). For these cancers, instead, we visited the GEPIA2 (Gene Expression Profiling Interactive Analysis 2nd Edition) web server (http://gepia2.cancer-pku.cn/#analysis) (Tang et al., 2019) to gain boxplots showing the expression differences between tumor tissues and their matching normal tissues from the GTEx (Genotype–Tissue Expression) database, with the main parameters set as follows: *p*-value cutoff at 0.01 and absolute log2 fold change cutoff at 1 and expressing as “Match TCGA normal and GTEx data” (Cui X. et al., 2020; Shen Y.-T. et al., 2021). Furthermore, we obtained violin plots of the KDM6B expression across all types of tumors at different pathological stages (stage I to IV) in TCGA conducted by one-way ANOVA with the “pathological stage plot” module. Log2 TPM (transcripts per million) + 1 transformed expression data were applied to convert the expression data in the box or violin graph, and Pr (>F) < 0.05 was considered to be statistically significant (Shen Y.-T. et al., 2021). Also, the Human Protein Atlas website (https://www.proteinatlas.org/) was used to explore the KDM6B levels across 20 tumor types by entering “KDM6B” (Uhlén et al., 2015).

### Analysis of Survival Prognosis

We employed GEPIA2 to acquire significance map data showing the OS (overall survival) and DFS (disease-free survival) outcomes of high and low KDM6B expressions across all tumors in TCGA. As previously reported, cutoff-high (50%) and cutoff-low (50%) values were applied as thresholds to divide the samples into the high- and low-expression cohorts (Cui X. et al., 2020; Zhao et al., 2020; Wu et al., 2021). We also obtained survival plots and performed a hypothesis test *via* the log-rank test. Moreover, we used the Kaplan–Meier plotter web server (http://kmplot.com/analysis/) to perform an analysis of OS, DMFS (distant metastasis-free survival), FP (first progression), PFS (progress-free survival), RFS (relapse-free survival), and PPS (post-progression survival) (Lánczky and Győrffy, 2021). As previously reported, the patients of breast, ovarian, gastric, and lung cancers were divided into two groups by the median expression level of KDM6B. Hazard ratio (HR) with 95% confidence intervals and the corresponding log-rank *p* value were computed, and then, the K–M survival plots were drawn (Hou et al., 2017; Lei et al., 2021).

### Analysis of Genetic Alteration and Methylation Modification

We tested KDM6B genetic change features in the cBioPortal web (https://www.cbioportal.org/) (Szklarczyk et al., 2015; Zhou et al., 2019). After that, we acquired information on the frequency of changes, type of mutation, and copy number changes in all tumors from TCGA. Furthermore, we acquired information in overall survival across all TCGA tumors followed with KDM6B gene alterations or not. Furthermore, the Kaplan–Meier plots that were generated with *p* log-rank values and *p* value < 0.05 were considered significant. Moreover, we selected the MEXPRESS website (https://mexpress.be/) (Koch et al., 2015) to investigate the KDM6B DNA methylation level of multiple probes across different tumors in TCGA datasets. The beta value, Benjamini–Hochberg-adjusted *p*-value, and Pearson correlation coefficient (R) value were calculated. We also analyzed the relationships between the expression of KDM6B and four DNA methyltransferases [DNMT1, DNMT2 (TRDMT1), DNMT3A, and DNMT3B] with TIMER2.0 (Yan et al., 2021). Heatmap colors represent the purity-adjusted partial Spearman’s rho value.

### Analysis of Immune Infiltration

We explored the association between the KDM6B expression and immune infiltration across all tumors in TCGA with TIMER2.0. CIBERSORT, CIBERSORT-ABS, TIMER, quanTIseq, MCP-counter, xCell, and EPIC algorithms (Hao et al., 2021) were applied to evaluate the immune infiltration data in all tumors across all immune cells in TIMER2.0, including monocytes, mast cells, macrophages, CD4^+^ T cells, CD8^+^ T cells, Treg, follicular helper T cells, NK T cells, NK cells, neutrophils, common lymphoid progenitors, hematopoietic stem cells, common myeloid progenitors, endothelial cells, DCs (dendritic cells), granulocyte–monocyte progenitors, myeloid-derived suppressor cells, eosinophils, and cancer-associated fibroblasts. The purity-adjusted Spearman’s rank correlation test was used to obtain the *p*-values and sectional correlation values. Finally, the data across all the immune cells were shown in the form of heatmaps, and the data on cancer-associated fibroblasts were also visualized as scatterplots.

### Correlation of the KDM6B Expression With TMB (Tumor Mutation Burden), MSI (Microsatellite Instability), and MMR (Mismatch Repair)

TMB represented Mut/Mb (the number of mutations per mega base) of DNA in tumor cells (Addeo et al., 2021). MSI resulted from mismatch repair deficiency and was correlated with a favorable prognosis compared with microsatellite stable cancers (Dan et al., 2019). TMB and MSI scores were calculated with R based on the data downloaded from TCGA (Cheng et al., 2021). Spearman correlation analysis was performed to investigate the relationship between the KDM6B expression and TMB and MSI. MMR was a DNA repair mechanism which repaired the mismatched nucleotide bases to normal. Thus, we explored the potential association between the level of KDM6B and MMR genes (MLH1, MSH2, MSH6, PMS2, and EPCAM) using TIMER2.0, with the heatmap colors representing the partial Spearman’s rho value.

### Analysis of KDM6B-Related Gene Enrichment

We visited the functional protein association networks’ (STRING) web server (https://string-db.org/) (Szklarczyk et al., 2015) with the basic parameters defined as follows: minimum required interaction score (“low confidence 0.150”), maximum number of interactors to display (“no more than 50 interactors” in the first shell), meaning of the network edges (“evidence”), and active interaction sources (“experiments”), as previously stated (Li et al., 2021). Finally, we acquired the top 50 KDM6B-related proteins with experimental evidence. With Cytoscape software (Shannon et al., 2003), we obtained the protein–protein interaction network of these proteins. The GEPIA2 “Similar Gene Detection” module was applied to recognize the top 100 KDM6B-related target genes from all tumor tissues in the TCGA dataset. To explore the correlation between KDM6B and the chosen genes, the Pearson correlation analysis on the paired genes was performed. Scatter plots with log2 (TPM) were drawn to determine and show the coefficient of correlation (R-value) and *p*-value (Cui X. et al., 2020). Moreover, with top 50 KDM6B-related proteins, we transferred gene symbol ID to Entrez ID with org.Hs.eg.db (version 3.10.0) and then performed the KEGG (Kyoto encyclopedia of genes and genomes) and GO (Gene ontology) analyses and visualized the results with “clusterProfiler” (version 3.14.3) and “gplot2” (version 3.3.3) R packages (Yu et al., 2012), with the two-tailed *p* value <0.05 considered statistically significant.

## Results

### KDM6B Expression in Cancers

**FIGURE 1:**
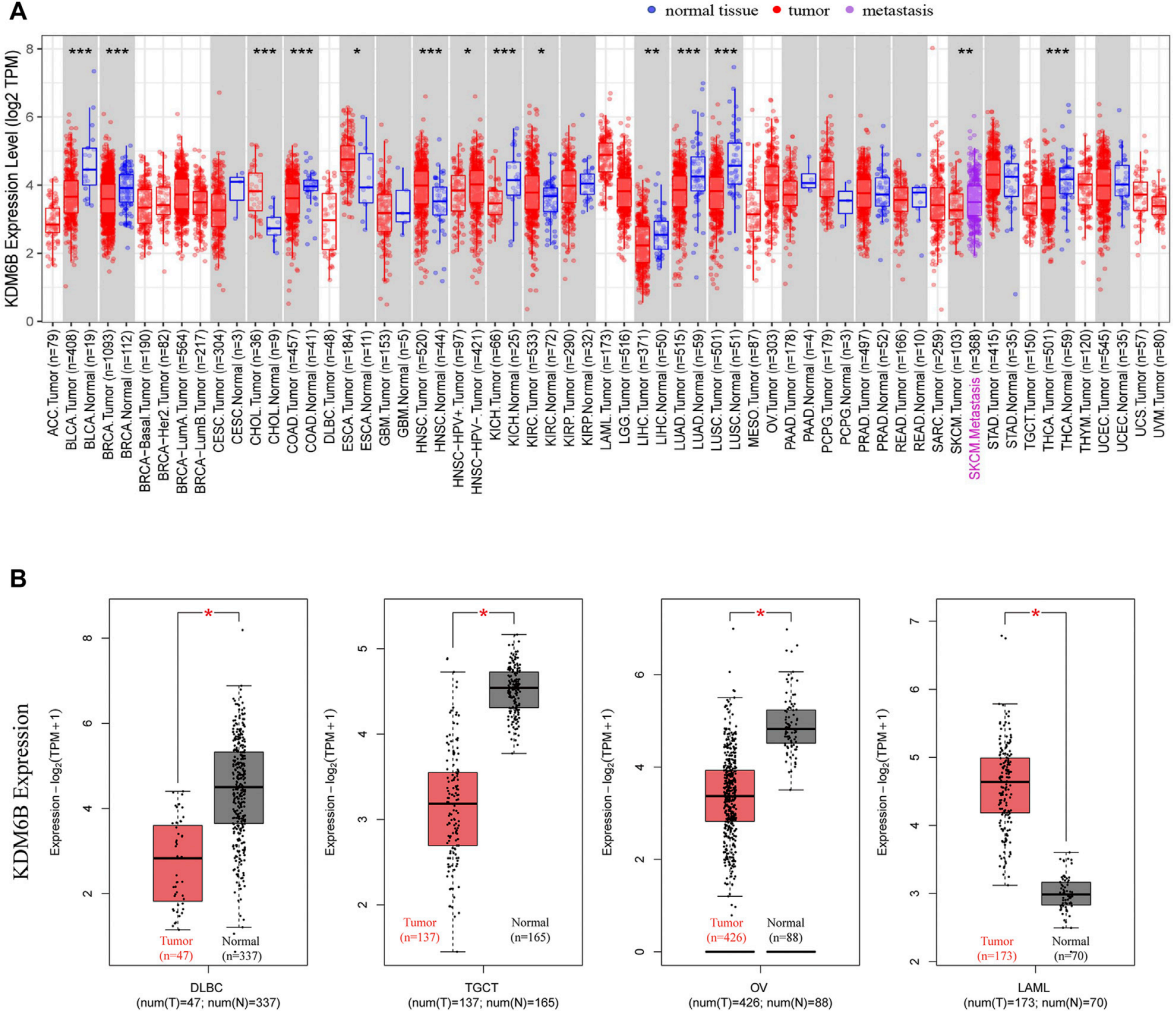
Expression of KDM6B in different tumors. **(A)** Expression status of KDM6B in different cancers or specific cancer subtypes was analyzed through TIMER2. *p < 0.05; **p < 0.01; ***p < 0.001. **(B)** For the types of DLBC, LAML, OV, and TGCT in the TCGA project, the corresponding normal tissues from the GTEx database were included as controls. The box plot data were supplied. *p < 0.05.

Using TIMER2, we investigated the KDM6B expression levels across all tumors in TCGA. As reported (Cui X. et al., 2020), we used normal tissues from GTEx data to assess the KDM6B expression difference between tumor and normal tissues when extremely limited normal tissue samples were obtained in TCGA, and the results were exhibited against a white background in Figure 1A. As demonstrated in Figure 1A, KDM6B shared a lower expression level in the tumor tissues of BLCA (bladder urothelial carcinoma), BRCA (breast invasive carcinoma), COAD (colon adenocarcinoma), KICH (kidney chromophobe), LUAD (lung adenocarcinoma), LUSC (lung squamous cell carcinoma), THCA (thyroid carcinoma) (all *p* < 0.001), and LIHC (liver hepatocellular carcinoma) (*p* < 0.01). On the contrary, the KDM6B expression level increased in the tumor tissues of CHOL (cholangiocarcinoma), HNSC (head and neck squamous cell carcinoma) (both *p* < 0.001), ESCA (esophageal carcinoma) and KIRC (kidney renal clear cell carcinoma) (both *p* < 0.05). For DLBC (lymphoid neoplasm diffuse large B-cell lymphoma), LAML (acute myeloid leukemia), OV (ovarian serous cystadenocarcinoma), and TGCT (testicular germ cell tumors), the KDM6B level decreased in the tumor tissues of DLBC, TGCT, and OV, while KDM6B expressed higher levels in the tumor tissues of LAML, all with *p* < 0.01 (Figure 1B).

**FIGURE 2:**
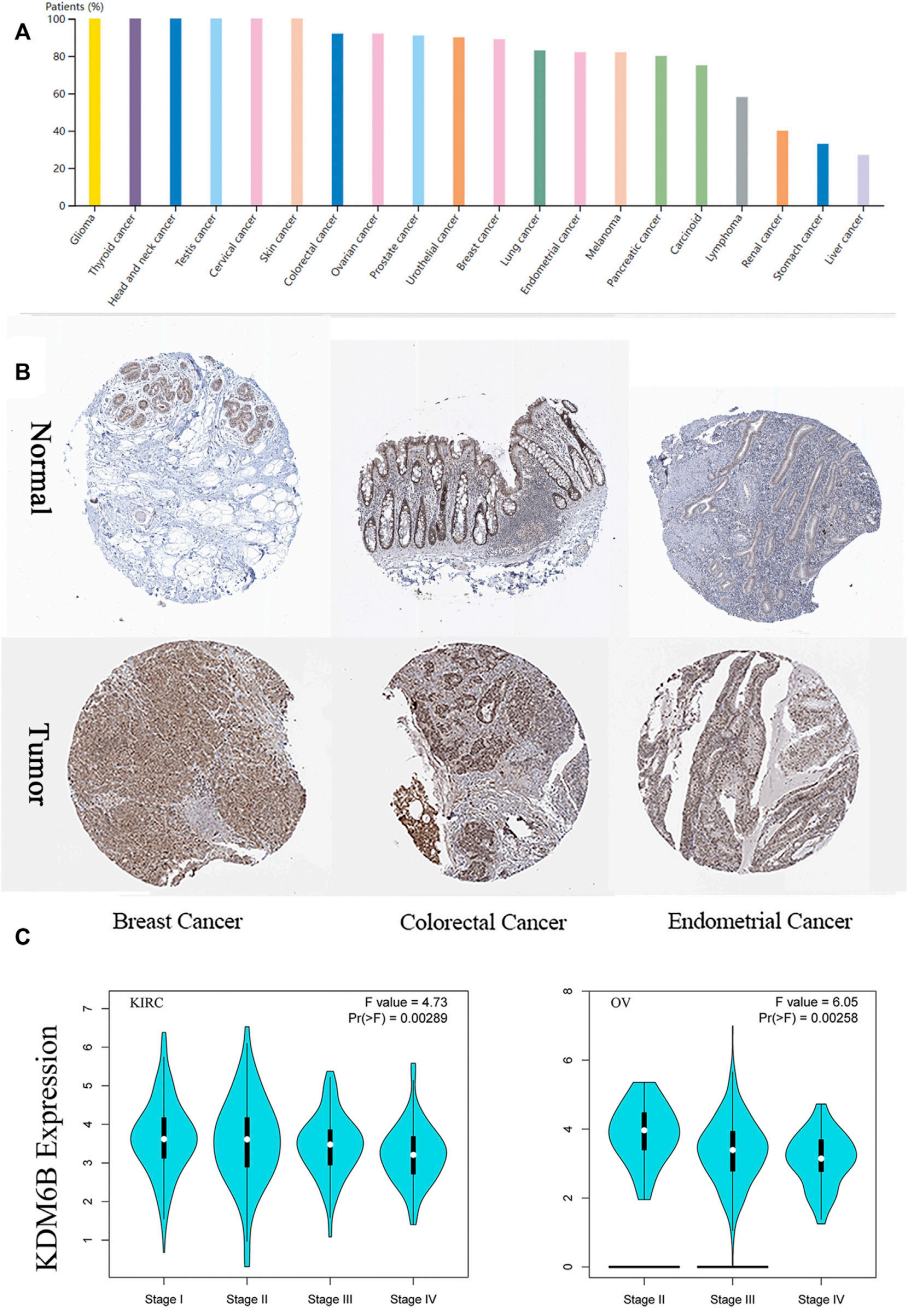
Expression of KDM6B in different tumors and pathological stages. **(A)** KDM6B protein level across 20 cancer types in Human Protein Atlas (HPA). **(B)** Representative immunohistochemical staining of KDM6B in HPA. **(C)** Based on the TCGA data, the KDM6B expression levels were analyzed in the main pathological stages (stage I, II, III, and IV) of KIRC, and OV. log2 (TPM + 1) was applied for log scale.

In addition, we explored the KDM6B protein expression in the cohort of the Human Protein Atlas database across 20 different tumor types. The results revealed that most malignant cells were moderately positive (Figure 2). Intense staining was sometimes seen in cervical, testicular, and endometrial cancers. Some cancers stained poorly or negatively, including hepatocellular, renal, and gastric cancers (Uhlén et al., 2015).

We also found that the KDM6B expression levels were significantly related to the pathological stages of KIRC and OV with GEPIA2 (Figure 2C). To describe the expression differences among different stages more accurately, we performed the Wilcoxon signed-rank test to analyze the KDM6B expression at different pathological stages of KIRC and OV with the TCGA portal website (http://tumorsurvival.org/), as previously reported (Shen E. et al., 2021). The *p*-value and box figures were exhibited in the (Supplementary Figure S1). The aforementioned results suggested that the KDM6B expression differed in cases with diverse types and stages of certain cancers.

### Survival Analysis

**FIGURE 3:**
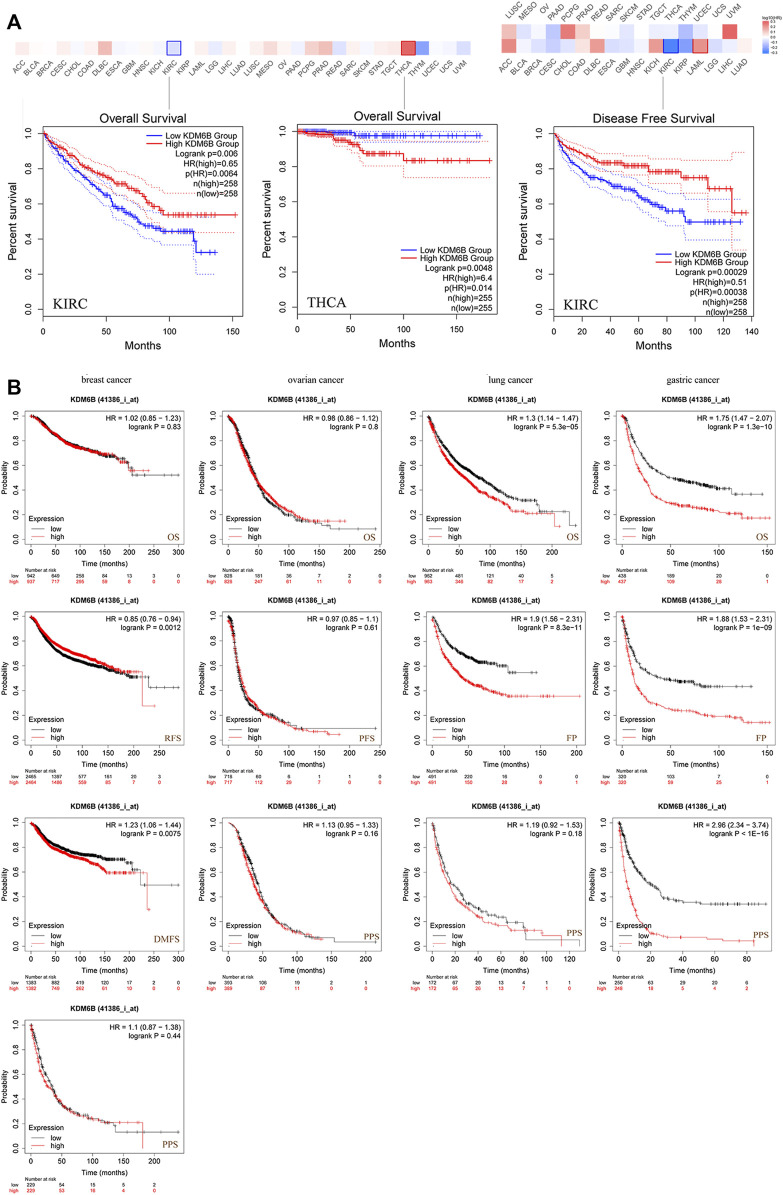
Correlation between the KDM6B expression and survival prognosis of cancers in TCGA. **(A)** Using the GEPIA2 tool to perform overall survival and disease-free survival analyses of different tumors in TCGA with the KDM6B expression. **(B)** Using the Kaplan–Meier plotter to perform a series of survival analyses, including OS, DMFS, RFS, PFS, PPS, and FP *via* the expressions of KDM6B in breast cancer, ovarian cancer, lung cancer, and gastric cancer cases.

We divided the cases into high and low KDM6B expressing groups and analyzed the association between the KDM6B expression level and clinical outcomes in different cancer cases. As shown in Figure 3A, highly expressed KDM6B was associated with the poor prognosis of OS for THCA (*p* = 0.0048). In addition, a low KDM6B expression was correlated with the poor OS prognosis (*p* = 0.006) and DFS prognosis (*p* = 0.00029) for KIRC. Moreover, we used the Kaplan–Meier plotter tool to explore the survival data on other cancers (Gyorffy, 2021). The results showed a relevance between a low KDM6B expression and poor RFS (Figure 3B, *p* = 0.0012) prognosis for breast cancer. However, a high KDM6B expression was correlated with a poor DMFS prognosis (*p* = 0.0075) for breast cancer (Figure 3B). On the contrary, high KDM6B expressions were related to poor OS (*p* = 5.3e-05) and FP (*p* = 8.3e-11) prognosis for lung cancer (Figure 3B) and poor OS (*p* = 1.3e-10), FP (P = 1e-09) and PPS (*p* < 1.0e-16) prognosis for gastric cancer (Figure 3B). Table 1 showed the effects of low KDM6B expression on clinical outcomes in various cancers based on our results.

**TABLE 1:** Effects of a low KDM6B expression on clinical outcomes in various cancers. The patient samples were divided into two cohorts based on the median expression (high-expression and low-expression group) of KDM6B. The “Expression Level” showed the expression difference in tumor tissues and matched normal tissues.

Expression level	Cancer type	Clinical outcomes					
		OS	DFS	FP	RFS	DMFS	PPS
Increased	Kidney renal clear cell carcinoma	Worse	Worse	—	—	—	—
Decreased	Thyroid carcinoma	Better	—	—	—	—	—
Decreased	Breast cancer	—	—	—	Worse	Better	—
Decreased	Lung cancer	Better	—	Better	—	—	—
—	Gastric cancer	Better	—	Better	—	—	Better

OS (overall survival), DFS (disease-free survival), FP (first progression), RFS (relapse-free survival), DMFS (distant metastasis-free survival), and PPS (post-progression survival).

### Genetic Alteration and DNA Methylation Modification Analysis

**FIGURE 4:**
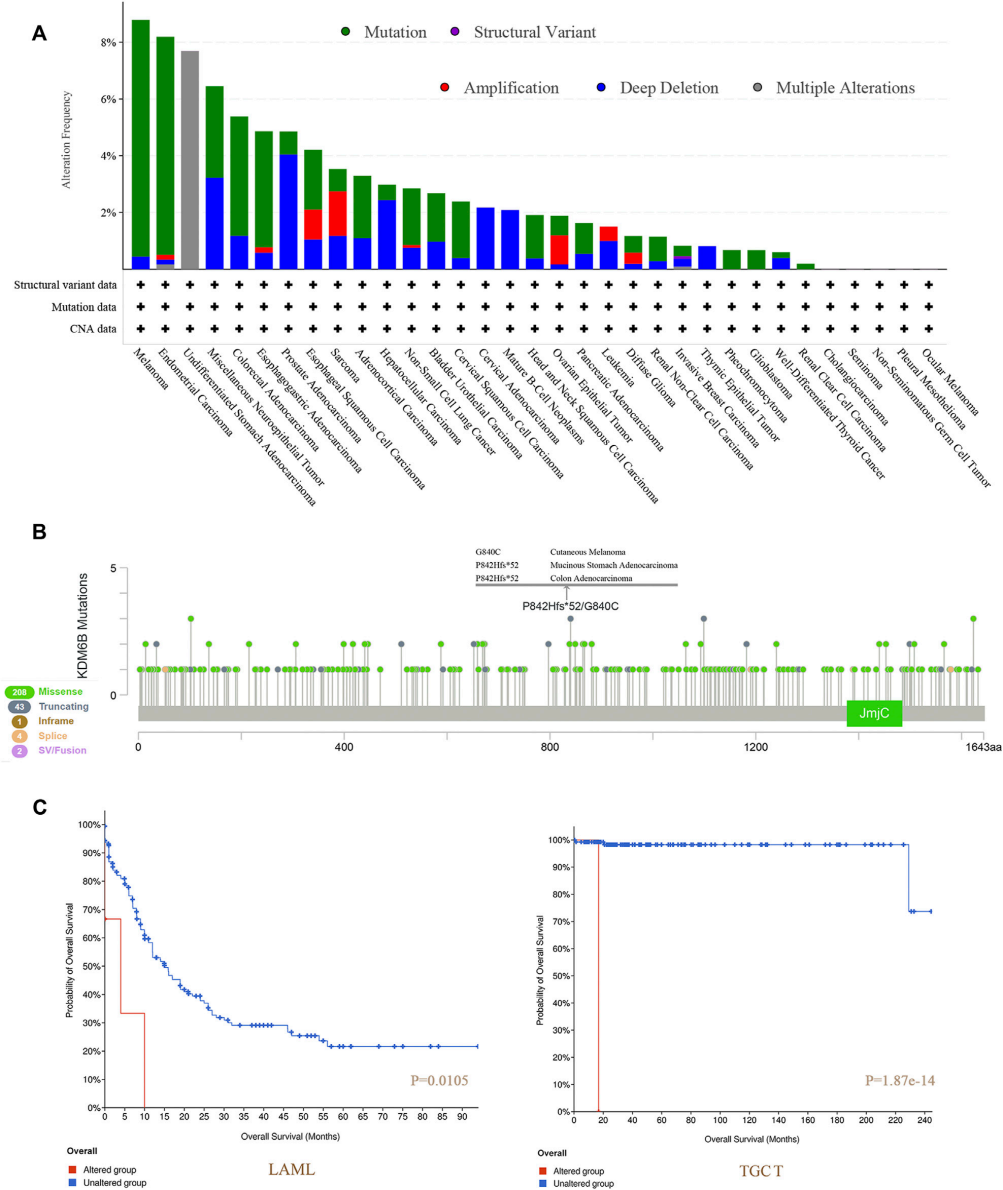
Mutation features of KDM6B in different tumors of TCGA. We analyzed the mutation features of KDM6B for the tumors in TCGA dataset using the cBioPortal tool. The alteration frequencies with mutation type **(A)** and mutation site **(B)** are displayed. We also analyzed the potential correlation between the mutation status and overall survival of LAML and TGCT **(C)** using the cBioPortal tool.

We detected a genetic change of KDM6B in several tumor cases from TCGA projects with cBioPortal. The highest frequency of change in KDM6B (>8%) was found in melanoma patients with “mutation” as the main type (Figure 4A). The “multiple alterations” composed of all types of the undifferentiated stomach adenocarcinoma cases, which showed an alteration frequency of >7% (Figure 4A). Most prostate adenocarcinoma cases with genetic changes had an altered copy number deletion of KDM6B (∼4% frequency) (Figure 4A). Notably, the “amplification” type was the predominant type of sarcoma cases, with a change rate of approximately 2% (Figure 4A). The KDM6B mutation type, location, and number of cases are shown in Figure 4B. Moreover, we investigated the associations between changes in the KDM6B gene and clinical outcomes in different cancers. The data in Figure 4C indicate that the cases without altered KDM6B showed better OS when compared with those with KDM6B alteration in LAML (*p* = 0.0105) and TGCT (*p* = 1.87e-14).

Next, as shown in Supplementary Figure S2, we tested the relationship between the KDM6B level and DNA methyltransferases, including DNMT1, DNMT2 (TRDMT1), DNMT3A, and DNMT3B with TIMER2.0. The results revealed that the KDM6B expression level was positively related to DNA methyltransferases in various cancers significantly, especially in BRCA−Basa, BRCA−Her2, BRCA−LumA, CESC, DLBC, ESCA, KIRP, LGG, LIHC, and THYM. With the TCGA project, we applied the MEXPRESS to explore the possible links of KDM6B and DNA methylation to the differential pathogeneses of cancer. For TGCT, we detected that methylation of KDM6B DNA was significantly correlated with a multi-probe gene expression in the non-promoter regions, such as cg19449286 (*p* < 0.001 and R = 0.464), as shown in Supplementary Figure S1B.

### Immune Infiltration Analysis

**FIGURE 5:**
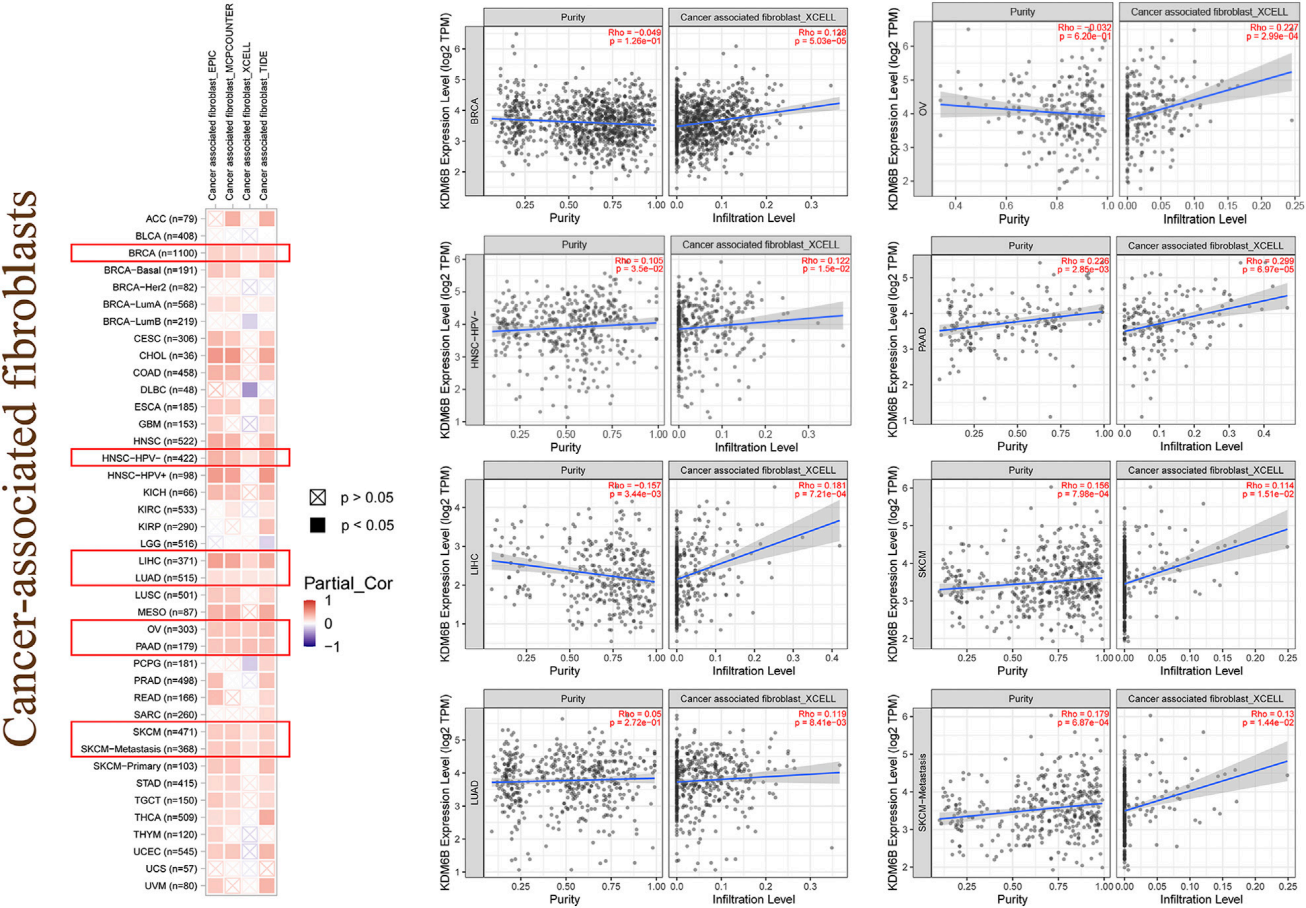
Immune infiltration of cancer-associated fibroblasts. Different algorithms were used to explore the potential correlation between the expression level of the KDM6B and the infiltration level of cancer-associated fibroblasts across all types of cancers in TCGA.

Tumor-infiltrating immune cells, a significant part of the microenvironment in a tumor, were deeply linked to the tumor origin, progression, and metastasis (DeBerardinis, 2020). It has been conveyed that tumor-associated fibroblasts, which comprised the microenvironment of the tumor, played a role in immune infiltration regulation of various immune cells in tumors (Fridman et al., 2011; Steven and Seliger, 2018; Chen and Song, 2019). In this study, we assessed the potential association between the immune infiltration level and KDM6B expression across different tumors in TCGA with the CIBERSORT, CIBERSORT-ABS, TIMER, xCell, MCP-counter, quanTIseq, and EPIC algorithms. KDM6B was associated with multiple immune cells, suggesting that KDM6B may affect the progression and clinical outcomes of tumors *via* the tumor microenvironment. Analysis executed with all or most of the above-mentioned algorithms showed significantly positive correlations between immune infiltration and KDM6B expression in different cancers, for example, Tregs and COAD; endothelial cell and COAD, KIRC, PAAD, SKCM-metastasis, and STAD; neutrophils and PCGC, PRAD, and THCA; and follicular helper T cells and GBM and UCEC (Supplementary Figure S3, Table 2). However, the immune infiltration of T-cell gamma delta was negatively correlated with the KDM6B expression in BRCA-LumA and CESC (Supplementary Figure S2, Table 2). In addition, we detected a significantly positive association between the KDM6B expression and tumor-associated fibroblast infiltration levels in tumors of BRCA, HNSC−HPV−, LIHC, LUAD, OV, PAAD, SKCM, and SKCM−Metastasis from TCGA (Figure 5).

**TABLE 2:** KDM6B expression level and the correlation with immune infiltration, TMB, and MSI in various cancers, in which the KDM6B expression level was significantly different from its matched normal tissues. *p* < 0.05 was considered significant, and only the significant correlation was exhibited in the table.

Expression level	Cancer type	Immune infiltration		TMB	MSI
		Positive	Negative		
Low	BLCA (bladder urothelial carcinoma)	Mast cells and neutrophils	Macrophages and monocytes	—	—
	BRCA (breast invasive carcinoma)	Cancer-associated fibroblasts and mast cells	T-cell gamma delta	-0.197	—
	COAD (colon adenocarcinoma)	Tregs, B cells, cancer-associated fibroblasts, dendritic cells, endothelial cells, macrophages, monocytes, and neutrophils	—	0.118	0.107
	KICH (kidney chromophobe)	Mast cells, monocytes, cancer-associated fibroblasts, and neutrophils	—	—	—
	LUAD (lung adenocarcinoma)	B cells, cancer-associated fibroblasts, and endothelial cells	Dendritic cells	—	0.268
	LUSC (lung squamous cell carcinoma)	Tregs, cancer-associated fibroblasts, endothelial cells, and mast cells	Dendritic cells and CD8^+^ T cells	—	0.414
	THCA (thyroid carcinoma)	Endothelial cells, cancer-associated fibroblasts, mast cells, and neutrophils	—	—	—
	LIHC (liver hepatocellular carcinoma)	CD4^+^ T cells, cancer-associated fibroblasts, tregs, B cells, monocytes, and neutrophils	—	−0.263	—
	DLBC (lymphoid neoplasm diffuse large B-cell lymphoma)	—	—	—	−0.562
	OV (ovarian serous cystadenocarcinoma)	Cancer-associated fibroblasts	CD8^+^ T cells and dendritic cells	—	—
	TGCT (testicular germ cell tumors)	Tregs, macrophages, and cancer-associated fibroblasts	—	—	—
High	CHOL (cholangiocarcinoma)	Cancer-associated fibroblasts	—	—	—
	HNSC (head and neck squamous cell carcinoma)	Endothelial cells, neutrophils, and cancer-associated fibroblasts	CD8^+^ T cells	0.139	—
	ESCA (esophageal carcinoma)	NK cells and cancer-associated fibroblasts	—	—	—
	KIRC (kidney renal clear cell carcinoma)	Endothelial cells, mast cells, monocytes, neutrophils, and NK cells	—	—	—
	LAML (acute myeloid leukemia)	—	—	—	—

### Analysis of TMB, MSI, and MMR

**FIGURE 6:**
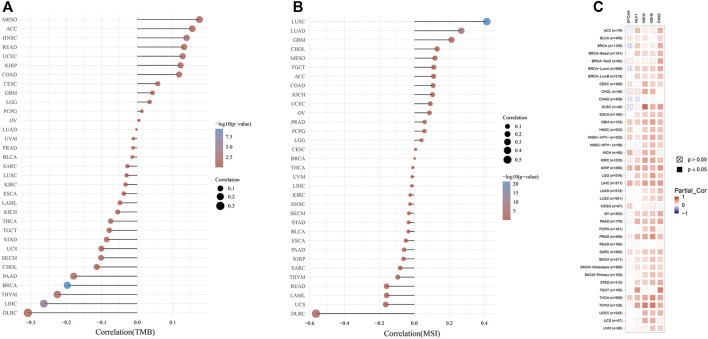
Correlations between the KDM6B expression and tumor mutational burden (TMB), microsatellite instability (MSI), and mismatch repair (MMR). **(A)** Spearman correlation analysis of KDM6B and TMB. **(B)** Spearman correlation analysis of KDM6B and MSI. The abscissa represents the correlation coefficient between genes and TMB or MSI; the ordinate represents different tumors. The size of the dots represents the size of the correlation coefficient, and the colors represent the significance of the *p* value. The bluer the color, the smaller is the *p* value. **(C)** Correlations between KDM6B levels and MMR gene (*MLH1, MSH2, MSH6, PMS2,* and *EPCAM*) expressions.

As exhibited in Figure 6A, the KDM6B expression showed a significantly positive correlation with TMB in HNSC, UCEC, COAD, and KIRP, whereas a higher expression of KDM6B was negatively correlated with TMB in BRCA, LIHC, THYM, PAAD, and SKCM. A similar relationship was found existing between KDM6B and MSI. As exhibited in Figure 6B, KDM6B was significantly related to six types of cancers, including LUSC, LUAD, DLBC, GBM, COAD, and UCEC, among which the majority of the associations were positive, except DLBC as the only type negatively correlated with KDM6B. The exact data on TMB and MSI are provided in Supplementary Table S2. Table 2 shows the correlation between the KDM6B expression level, TMB, and MSI in various cancers, in which the KDM6B expression level was significantly different with its matched normal tissues. As for the MMR genes, KDM6B was positively correlated with all five MMR genes in GBM, HNSC-HPV-, KIRC, KIRP, LIHC, PRAD, SARC, and THCA, whereas COAD was the sole type that only had a negative correlation with KDM6B (Figure 6C).

### KDM6B-Related Protein Enrichment Analysis

**FIGURE 7:**
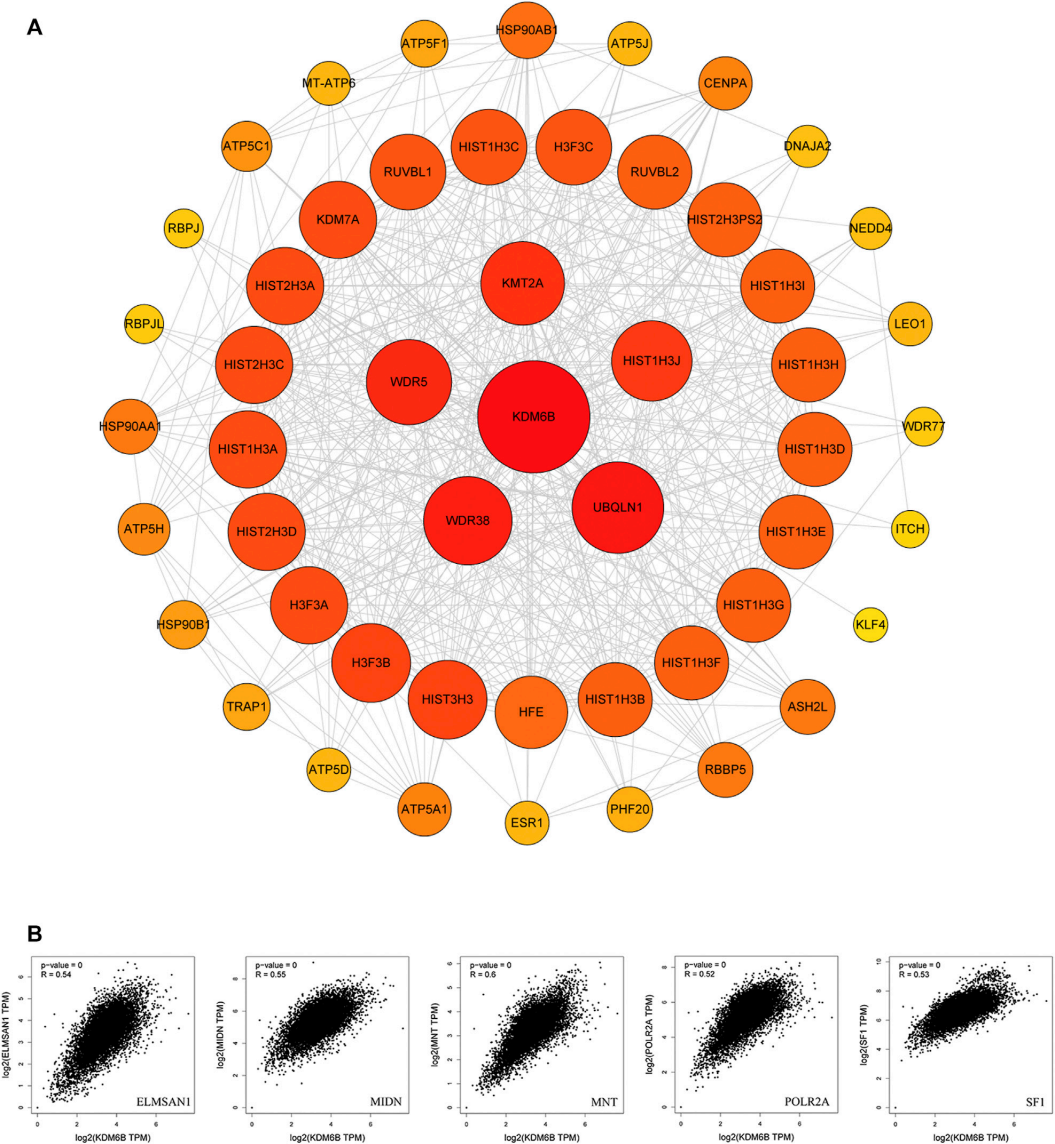
KDM6B-related gene enrichment analysis. **(A)** Protein–protein interaction (PPI) network of the top 50 available experimentally determined KDM6B-binding proteins obtained from the STRING website. **(B)** Using the GEPIA2 approach, we also obtained the top 100 KDM6B-correlated genes in TCGA projects and analyzed the expression correlation between KDM6B and related genes, including *ELMSAN1*, *MIDN*, *MNT*, *POLR2A*, and *SF1*.

To explore the potential mechanisms of KDM6B in tumor pathophysiology, we sought to highlight KDM6B-binding proteins and KDM6B expression-related genes in several pathway enrichment analyses. We gained top 50 experimentally determined KDM6B-binding proteins with STRING. The protein–protein interaction network of these proteins is shown in Figure 7A. With the GEPIA2 tool, we pooled all TCGA tumor expression data to gain the top 100 genes related to the KDM6B expression. As shown in Figure 7B, the KDM6B expression level was positively related to that of ELMSAN1 (MIDEAS, Mitotic deacetylase–associated SANT domain protein) (R = 0.54), MIDN (midnolin) (R = 0.55), MNT (MAX network transcriptional repressor) (R = 0.6), POLR2A (RNA polymerase II subunit A) (R = 0.52), and SF1 (splicing factor 1) (R = 0.53) genes (all *p* < 0.001).

**FIGURE 8:**
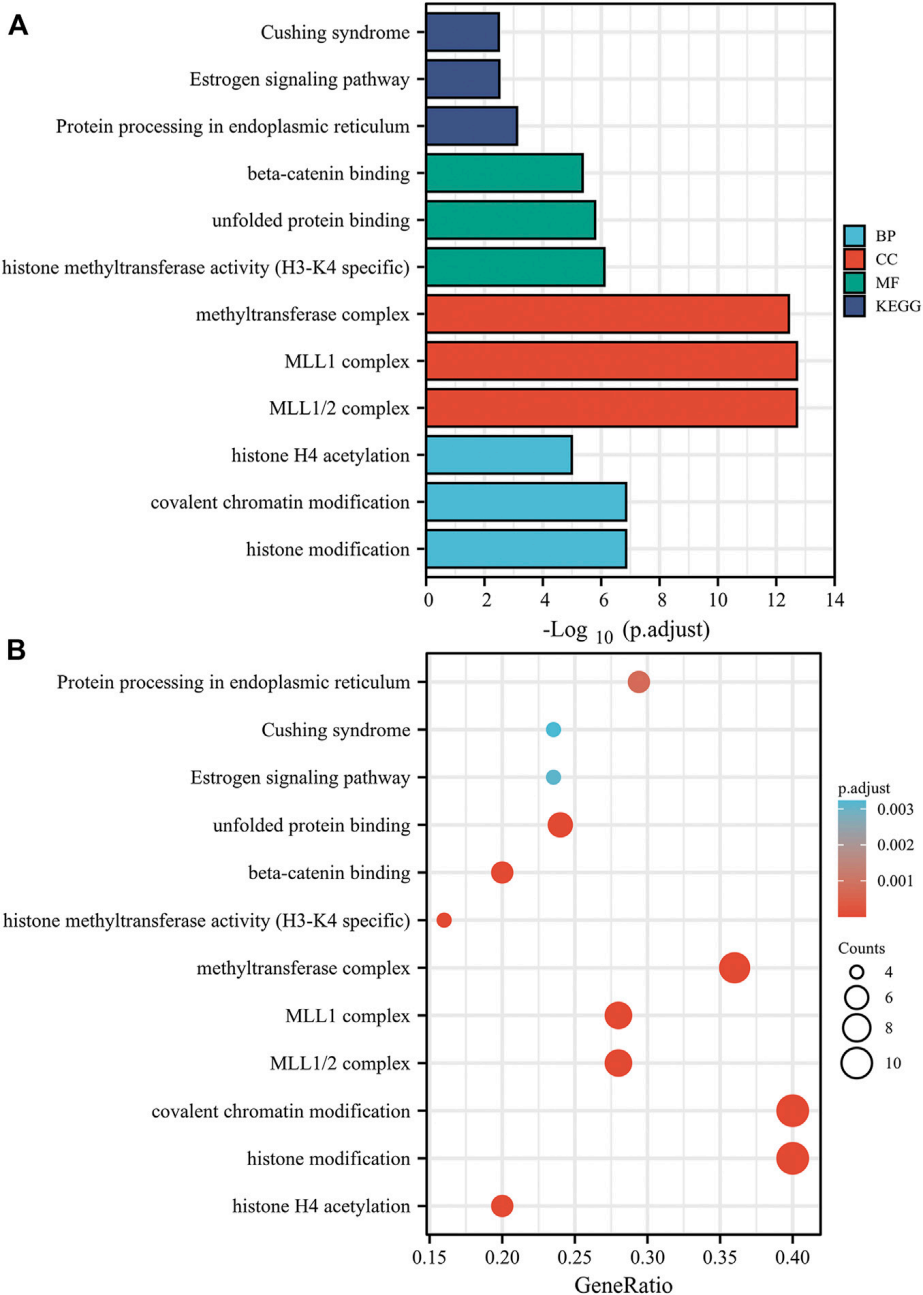
Based on the KDM6B-binding and interacted proteins, the KEGG pathway and GO analysis was performed and shown as bar chart **(A)** and bubble chart **(B)**. The top three significant terms in BPs (biological processes), MFs (molecular functions), and CCs (cellular components) and KEGG pathways are shown in bar chart. The GO analysis revealed that target proteins were mostly enriched in covalent chromatin modification, histone modification, and histone H4 acetylation in the BP enrichment analysis; methyltransferase complex, MLL1 complex, and MLL1/2 complex in the CC analysis; and histone methyltransferase activity (H3-K4 specific), modification-dependent protein binding, and unfolded protein binding in the MF analysis. The KEGG analysis indicated that the target proteins were significantly enriched in pathways of Cushing syndrome, estrogen signaling pathway, and protein processing in the endoplasmic reticulum.

Furthermore, we executed GO and KEGG analyses with the KDM6B-related proteins. The top three significant terms in BPs (biological processes), MFs (molecular functions), and CCs (cellular components) and KEGG pathways are shown in Figure 8. Detailed results of the GO and KEGG analyses are shown in Supplementary Table S2. The GO analysis revealed that target proteins were mostly enriched in covalent chromatin modification, histone modification, and histone H4 acetylation in BP enrichment analysis; methyltransferase complex, MLL1 complex, and MLL1/2 complex in CC analysis; and histone methyltransferase activity (H3-K4 specific), beta-catenin binding, and unfolded protein binding in MF analysis. The KEGG analysis indicated that target proteins were meaningfully enriched in pathways in Cushing syndrome, estrogen signaling pathway, and protein processing in the endoplasmic reticulum (Figure 8). These data suggested that histone methylation dysregulation may be associated with the role of KDM6B during tumor pathogenesis, which is consistent with previous studies (Qin et al., 2021; Yildirim-Buharalioglu, 2022).

Above all, the results showed that the KDM6B expression had a different impact on the prognosis among various cancers. Genetic alteration of KDM6B also affected the prognosis of acute myeloid leukemia (LAML) and testicular germ cell tumors (TGCTs). The KDM6B expression level was associated with DNA methyltransferase activity, TMB, MSI, and immune cell infiltration, across various cancer types with different correlations, through which KDM6B may impact on the clinical outcomes and immune therapeutic effects. The top 50 proteins most correlated were recognized using STRING. GO and KEGG enrichment analyses showed that the most closely related pathway was histone methylation regulation.

## Discussion

As a histone demethylase, KDM6B is responsible for the enzymatic removal of the repressive chromatin mark H3K27me3 which is involved in biological pathways such as differentiation, development, and apoptosis. These biological pathways tend to impact response reactions against intracellular or extracellular stimulations and suggest a functional link between KDM6B and clinical diseases, including inflammations, especially, cancers (Zhang et al., 2018; Mallaney et al., 2019; Cao et al., 2021). However, whether KDM6B has influences on the pathogenesis of various tumors *via* certain pathways remains unclear. This study intended to explore the dual role of KDM6B widely. Therefore, with the TCGA and GEO databases, we thoroughly characterized the KDM6B expression in 33 types of tumors and examined the related molecular properties of the expression level, genetic alterations, or DNA methylation. We found that the expression of KDM6B differed in different cancer types and stages. With the KM survival analyses, we revealed that an irregular KDM6B expression may serve as a prognostic biomarker in several cancers, such as breast cancer, lung cancer, and gastric cancer. Moreover, we also predicted that the KDM6B expression level was correlated with the tumor immune microenvironment. GO and KEGG analytic approaches were performed to recognize the KDM6B‐related biological mechanisms.

To improve the chances of cures for cancer patients, it is necessary to explore tumor-specific target molecules for a precise treatment through identifying the differentially expressed genes (Andre et al., 2014). Therefore, it was valuable to perform a pan‐cancer analysis of the expression difference and potential molecular mechanism of KDM6B across different cancers. The observation of KDM6B downregulation across cancer types was consistent with prior knowledge that KDM6B could regulate the expression levels of specific genes and the interactions of protein molecules contributing to tumor suppression (Lagunas-Rangel, 2021). In this work, we observed that KDM6B was down-regulated in 11 of 16 cancer types (Figure 1). On the contrary, we observed the KDM6B upregulation across 5 of 16 cancer types. Interestingly, we also observed that KDM6B upregulated in KIRC compared with the adjacent normal tissues, but the expression level gradually decreased as the tumor progressed. In tumorigenesis, KDM6B induced an epithelial−mesenchymal transition and lymph node metastasis in KIRC (Li et al., 2015). However, our results revealed that a higher expression level of KDM6B may be correlated with lower stages and favorable survival outcomes, which may be attributed to cell-type specific effects. Nonetheless, the role of KDM6B in these cancers, especially KIRC, still needed to be further explored.

Although immunotherapy has shown increasing the therapeutic impact in tumors, we barely obtained any reports focusing on the relationship between the KDM6B expression and tumor immunity through literature search. Therefore, we explored the potential association between KDM6B expression and immune infiltration levels of various immune cells with TIMER2. As a result, we detected a positive relation between the KDM6B expression and the immune infiltration level of Treg in COAD and LUSC; endothelial cell and COAD, KIRC, PAAD (pancreatic adenocarcinoma), SKCM (skin cutaneous melanoma)-metastasis, and STAD (stomach adenocarcinoma); neutrophil and PCGC (pheochromocytoma and paraganglioma), PRAD, and THCA; and follicular helper T cells and GBM (glioblastoma multiforme) and UCEC (uterine corpus endometrial carcinoma). Remarkably, for example, the immune infiltration of T-cell gamma delta was negatively correlated with the KDM6B expression in BRCA-LumA and CESC based on all or most algorithms. In addition, the results showed that the KDM6B expression correlates with the degree of the infiltration of tumor-associated fibroblasts in various cancers, including BRCA, HNSC (head and neck squamous cell carcinoma), HPV−, LIHC, LUAD (lung adenocarcinoma), OV, PAAD, SKCM, and SKCM−metastasis. Cancer-associated fibroblasts have been reported to have an important impact on tumor metastasis, progression, and prognosis (Houthuijzen and Jonkers, 2018; Paauwe et al., 2018). The evidence suggests the potential role KDM6B plays in the tumor immunity.

Generally, higher TMB was related to a favorable overall survival and better response to immunotherapy (Cui Y. et al., 2020). MSI status, resulting from defects in MMR, is likely to be an independent favorable predictor of survival in pan-cancer patients, with MSI-positive tumors having a better survival outcome (Weiss et al., 2011; Samstein et al., 2019). With the correlation varying in different tumors, the KDM6B expression level was correlated with TMB in nine cancer types and with MSI in six cancer types, thus affecting the response to immunotherapy. Our results also revealed that the KDM6B expression was positively related to the MMR gene expression in most tumors, except for COAD. Based on the previous studies, the aforementioned results suggested that KDM6B might affect the response to immunotherapy and survival outcomes in various cancer types *via* TMB, MSI, or MMR, offering a new predictor for the immunotherapy efficacy.

Induced by DNA methylases, characteristic hypermethylation in the promoter or enhancer of certain genes, especially cancer-associated genes, was a specific feature of cancer cells (Kondo et al., 2019). Based on the positive association between KDM6B and DNA methyltransferases in various cancers, we assumed that KDM6B may affect the DNA methylation status in tumorigenesis. Moreover, DNA methylation affected the KDM6B expression level as well, which in turn affected its function in cancers (Malouf et al., 2016; Luo et al., 2018). It was reported that KDM6B controls the spermatogonial compartment through the regulation of fragmentation of spermatogonial cysts (Iwamori et al., 2013). In TGCT patients, we detected a possible association between non-promoter DNA methylation and KDM6B expression, suggesting the possible impact of KDM6B DNA methylation on TGCT progression.

KDM6B plays oncogenic roles in several tumors. In prostate cancer, KDM6B demethylates H3K27me3 at cyclin D1 promoter and prompts the expression of cyclin D1 cooperating with smad2/3 (Cao et al., 2021). Upregulated KDM6B facilitates tumor metastasis in osteosarcoma through modulating the lactate dehydrogenase expression (Jiang et al., 2021). In addition to the demethylase activity, KDM6B upregulates the expression of the MAPK signaling pathway components including ELK1 and FOS and then mediates the growth and survival of multiple myeloma cells (Ohguchi et al., 2017). On the contrary, KDM6B can also serve as a tumor suppressor. In non-small cell lung cancer patients, KDM6B significantly decreases in the serum and may play a pro-apoptotic role *via* promoting the nuclear translocation of FOXO1 (Ma et al., 2015; Ge et al., 2019). In squamous cell carcinoma, KDM6B is repressed by the transcription factor CSL, contributing to tumor proliferation, occurrence, and correlated inflammation (Al Labban et al., 2018). In neuroblastoma, KDM6B is upregulated by retinoic acid *via* HOXC9 to remove the repressive chromatin marker H3K27me3 and induce neuronal differentiation, thus inhibiting cell proliferation and tumorigenicity (Yang et al., 2019). To explore the specific mechanisms of its dual effects in cancers, we used KDM6B-related proteins across all tumors to perform GO and KEGG enrichment analyses and identified the potential impact of H3K4 and H3K27 methylation on the pathogenesis of cancers. As one of the predominant epigenetic mechanisms, histone methylation has attracted increasing attention on its potential link with tumorigenesis in recent years (Fetahu and Taschner-Mandl, 2021). In addition to catalytically removing the methyl groups from H3K27me3, KDM6B also bonded to the Set1/MLL H3K4 methyltransferase complex to activate gene transcription (Shi et al., 2014; Yu et al., 2018). H3K4 trimethylation was associated with transcriptional activation, while H3K27 trimethylation contributed to transcriptional silencing (Lien et al., 2020). Hypermethylation of the proto-oncogene H3K4 may activate transcription of oncogenes and other cancer-associated genes, eventually contributing to the development and progression of tumors (Li et al., 2017). A similar effect can be achieved via upgraded methionine metabolism induced by H3K4 methylation (Tran et al., 2017). Furthermore, a meta-analysis revealed that a higher H3K4 trimethylation level was correlated with a poorer overall survival (Li et al., 2018). In epigenetic regulation for cancer occurrence and development, methylation of H3K27 was considered to be a precursor to abnormal methylation sites and commonly existed on tumor suppressor genes (Barzily-Rokni et al., 2011; Ryu et al., 2019). These pieces of evidence were consistent with the carcinogenic role of KDM6B in most tumors. Taking into account the transcriptional regulation mechanism, KDM6B can also achieve tumor suppression *via* the same way in certain cancers, such as neuroblastoma sphere-forming cells and squamous cell carcinoma cells. KDM6B upregulates the expression of specific genes, such as p53, p21, HOX, and ERβ so as to restrain cell growth and boost differentiation, senescence, and apoptosis through the histone demethylase activity in tumor cells (Lagunas-Rangel, 2021). The KEGG analysis also indicated that the crosstalk of the KDM6B and IL-17 signaling pathways may influence the effects of KDM6B in cancers (Supplementary Table S2). KDM6B, together with KDM6A, was identified as the core regulator of T helper (Th) cells, and the inhibition of KDM6A/B contributed to the metabolic reprogramming followed by the suppression of IL-17 levels in Th17 cells, which mainly produced IL-17 cytokine (Cribbs et al., 2020; Sugaya, 2020). Substantial evidence supported a promoting role for IL-17 in tumorigenesis. In prostate cancer, IL-17 promoted prostate carcinogenesis through the induction of epithelial-to-mesenchymal transition mediated by MMP7 (Zhang et al., 2017). In pancreatic ductal adenocarcinoma, IL-17 not only recruited neutrophils and activated neutrophil extracellular traps but also mediated the exclusion of cytotoxic CD8 T cells from tumors, with a higher expression of IL17 representing poorer clinical outcomes (Zhang et al., 2020). However, the anti-tumor effects of IL-17 in special conditions have also been revealed. IL-17A directly induced a decrease in differentiation, apoptosis, and proliferation of myeloid-derived suppressor cell lines, suggesting that increased IL-17 signaling may restore immune responses (Ma et al., 2018). In breast cancer, the antitumor effects of IL-17E, also namely IL-25 and a member of IL-17 family, were induced by apoptosis and infiltration of eosinophils and B cells *via* binding to IL‐25R on tumor cells (Gowhari Shabgah et al., 2021). Thus, the IL-17 signaling pathway may also participate in the dual role of KDM6B. Above all, KDM6B played dual roles in different oncological contexts *via* various mechanisms and its clinical significance remained to be further established.

Some limitations to this study should be addressed. The survival analysis in several cancers was not assessed due to a lack of available data. In addition, the functions of KDM6B in pan-cancers were only tested with the bioinformatics analysis; therefore, *in vitro* or *in vivo* experiments should be performed to conduct a more solid conclusion.

In summary, this study assessed the dual role of KDM6B in tumor progression and clinical outcomes across all types and stages of cancers in TCGA for the first time. KDM6B expression was associated with TMB, MSI, and immune cell infiltration, particularly cancer-associated fibroblasts. Thus, KDM6B may affect the response to immunotherapy and clinical outcomes. Dysregulation of the DNA methyltransferase activity and the methylation level of H3K4 and H3K27 involve in the dual role of KDM6B in tumorigenesis and development. It is of great value to investigate the exact role of KDM6B in tumorigenesis and immune microenvironment to generate more precise preventive measures and immunotherapy.

## Data Availability

Publicly available datasets were analyzed in this study. These data can be found at: The Cancer Genome Atlas (TCGA, https://tcga-data.nci.nih.gov/tcga/) and Gene Expression Omnibus (GEO, https://www.ncbi.nlm.nih.gov/geo/) databases.

## Supplementary Material

Supplementary Figure S1 Correlation between KDM6B expression and different stages of KIRC and OV.

Supplementary Figure S2 Association between KDM6B and DNA methylation.

Supplementary Figure S3 Correlation analysis between the KDM6B expression and immune infiltration with CIBERSORT, CIBERSORT-ABS, TIMER, xCell, McP-counter, quanTIseq, and EPIC algorithms. *p*-values <.05 were considered statistically significant.

Supplementary Table S1 Spearman correlation analysis between the KDM6B expression levels and tumor mutational burden (TMB) and microsatellite instability (MSI).

Supplementary Table S2 Detailed results of the GO and KEGG analyses of KDM6B-related proteins.
