# Supplementary figures and images for: A Pan-Cancer Analysis Revealing the Dual Roles of Lysine (K)-Specific Demethylase 6B in Tumorigenesis and Immunity
Source: Front Genet. 2022 Jun 14;13:912003. doi: 10.3389/fgene.2022.912003 (PMC9246050; doi:10.3389/fgene.2022.912003)

A

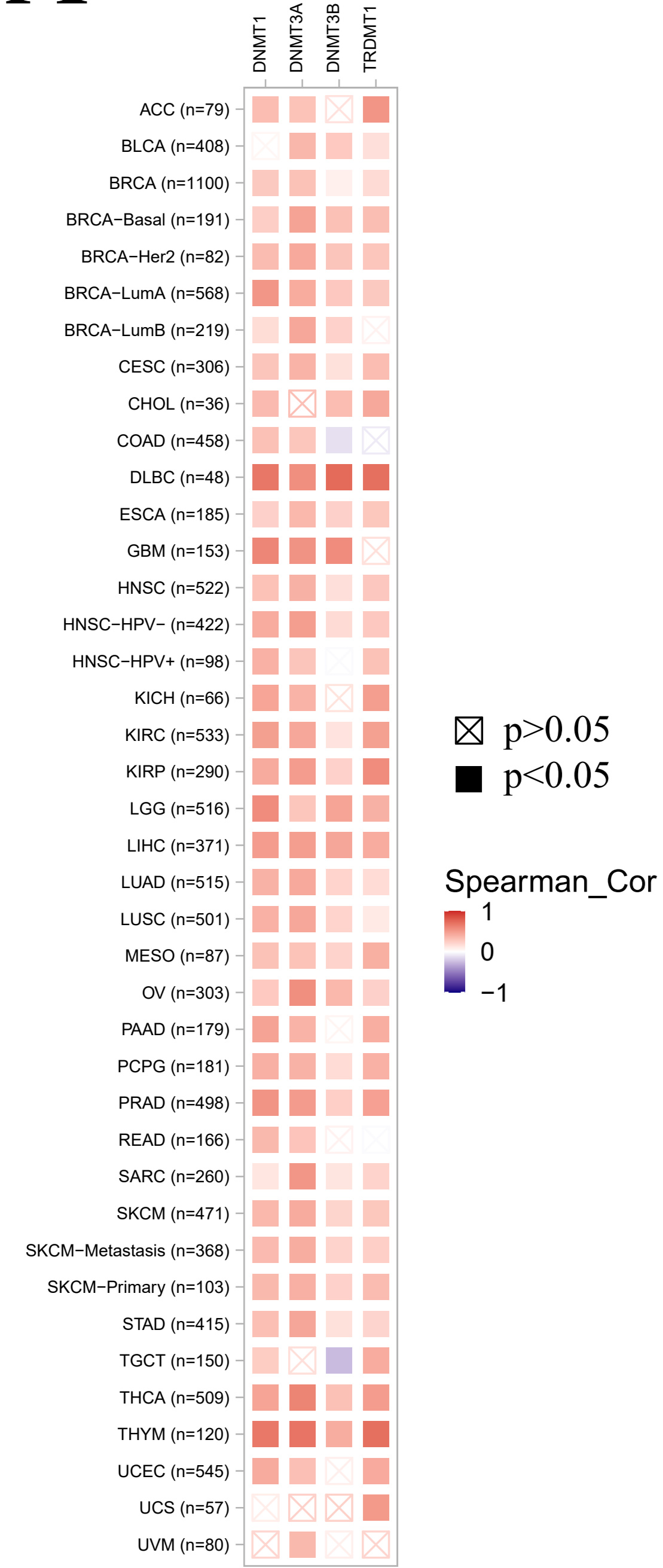

B

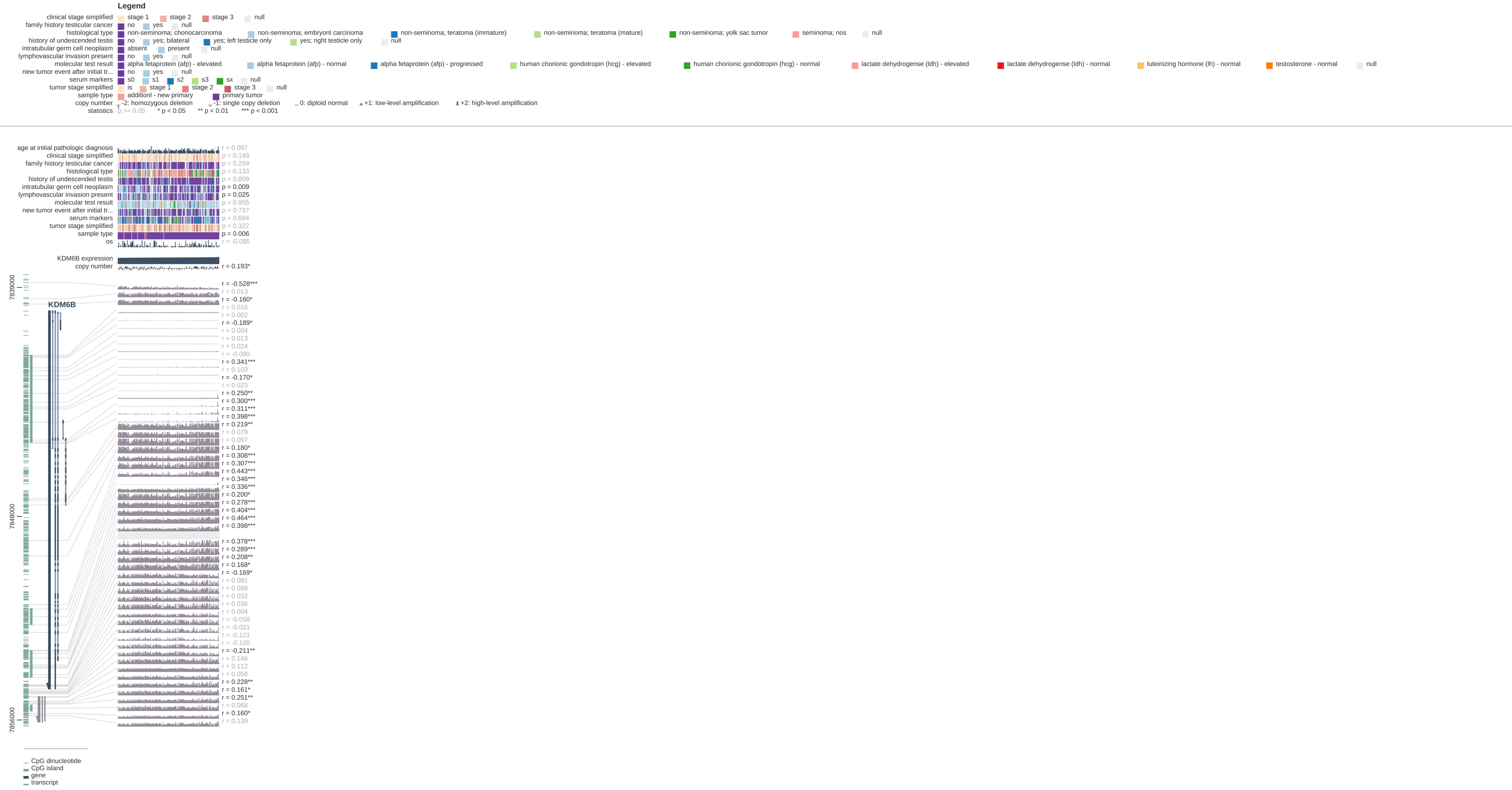

Supplement: Supplementary file 2 [file Image2.pdf]
