# Supplementary material for: A Pan-Cancer Analysis Revealing the Dual Roles of Lysine (K)-Specific Demethylase 6B in Tumorigenesis and Immunity
Source: Front Genet. 2022 Jun 14;13:912003. doi: 10.3389/fgene.2022.912003 (PMC9246050; doi:10.3389/fgene.2022.912003)
Supplement: Supplementary file 5 [file Image1.pdf]

A

	Stage I	Stage II	Stage III	Stage IV
Stage I	1.00	0.98	0.07	**0.00**
Stage II	0.98	1.00	0.30	**0.02**
Stage III	0.07	0.30	1.00	**0.03**
Stage IV	0.00	0.02	0.03	1.00

B

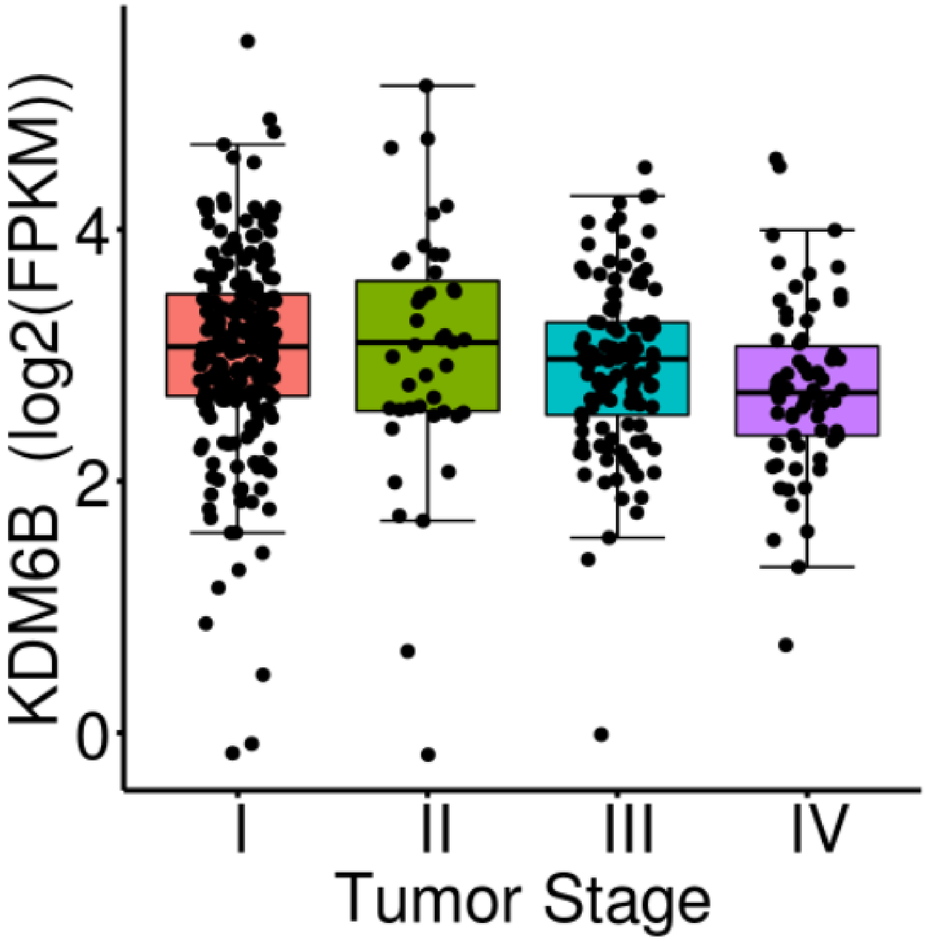

C

	IIA	IIB	IIC	IIIA	IIIB	IIIC	IV	NC
IIA	1.00	0.80	1.00	0.89	0.60	0.49	0.35	1.00
IIB	0.80	1.00	0.05	0.18	0.12	0.28	0.46	0.10
IIC	1.00	0.05	1.00	0.16	**0.02**	**0.01**	**0.00**	0.39
IIIA	0.89	0.18	0.16	1.00	0.74	0.69	0.26	0.18
IIIB	0.60	0.12	0.02	0.74	1.00	0.89	0.25	**0.02**
IIIC	0.49	0.28	0.01	0.69	0.89	1.00	0.13	0.08
IV	0.35	0.46	0.00	0.26	0.25	0.13	1.00	**0.02**
NC	1.00	0.10	0.39	0.18	0.02	0.08	0.02	1.00

D

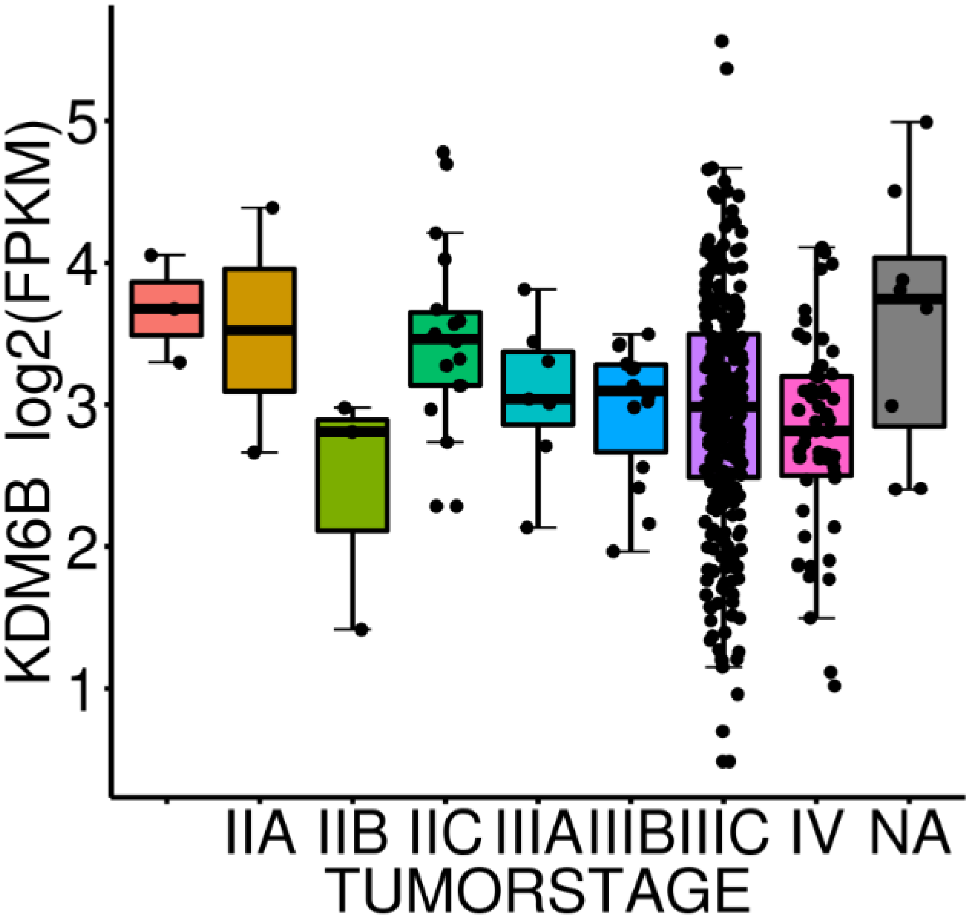

Figure S1. Correlation between KDM6B expression and different stages of KIRC and OV. (A) P value of Wilcoxon signed-rank test of KIRC. P value < .05 was considered statistically significant and highlighted with bold font. (B) Expression level of KDM6B gene in different pathological stages of KIRC. (C) P value of Wilcoxon signed-rank test of OV. P value < .05 was considered statistically significant and highlighted with bold font. (D) Expression level of KDM6B gene in different pathological stages of OV.
